# Herbal Compound “Jiedu Huayu” Reduces Liver Injury in Rats via Regulation of IL-2, TLR4, and PCNA Expression Levels

**DOI:** 10.1155/2017/9819350

**Published:** 2017-01-19

**Authors:** Minggang Wang, Qinglan Shi, Rongzhen Zhang, Hua Qiu, Dewen Mao, Fuli Long

**Affiliations:** Department of Liver Disease, The First Affiliated Hospital of Guangxi University of Traditional Chinese Medicine, Nanning, Guangxi 530023, China

## Abstract

*Aim of the Study*. To investigate the preventative effects of Jiedu Huayu (JDHY) on D-galactosamine (D-GalN) and lipopolysaccharide-induced acute liver failure (ALF) and to evaluate the possible mechanisms of action.* Materials and Methods*. ALF was induced in Wistar rats by administrating D-GalN (900 mg/kg) and lipopolysaccharide (10 *μ*g/kg). After treatment with JDHY granules, the levels of blood alanine aminotransferase, aspartate aminotransferase, total bilirubin, and prothrombin time were determined. Proliferating cell nuclear antigen was detected by immunohistochemistry staining. The expression of interleukin-2 (IL-2) and toll-like receptor 4 (TLR4) was examined by fluorescence quantitative reverse transcription polymerase chain reaction (qRT-PCR) and Western blot.* Results*. JDHY treatment dramatically improved liver function and increased survival rates in an ALF model in rats. We observed a decrease in IL-2 and TLR4 expression following treatment with JDHY in liver cells from ALF rats using qRT-PCR and Western blot analysis.* Conclusion*. We hypothesize that the therapeutic potential of JDHY for treating ALF is due to its modulatory effect on the suppression of inflammation and by promoting hepatocyte regeneration. Our results contribute towards validation of the traditional use of JDHY in the treatment of liver disease.

## 1. Introduction

Acute liver failure (ALF) is a life-threatening condition that is characterized by rapid deterioration of liver function in a short period of time. ALF typically presents clinical symptoms such as hepatic dysfunction, high jaundice, and coagulopathy. In addition, hepatic encephalopathy may occur within two weeks after the onset of the condition [1]. Despite the action of the liver support system and the improvement and advancement after liver transplantation, the mortality of ALF can reach as high as 50% [2]. The main reasons can be contributed to rapid disease progression and unpredictable outcomes of various factors [3]. In summary, there is a critical need for novel therapeutics to improve the curative effect.

Presently, the pathogenesis of ALF has not been clarified. Proliferating cell nuclear antigen (PCNA) can directly participate in DNA synthesis and strong expression of PCNA can be observed when DNA replication is activated [4]. It has previously been reported that increased PCNA expression can improve survival rate in ALF rats [5, 6]. Interleukin-2 (IL-2) is an important cytokine and is the key player in the transformation of precursor cells of cytotoxic T cell into cytotoxic T lymphocytes [7]. In addition, IL-2 is known to participate in the pathological progress of ALF [8]. Toll-like receptor 4 (TLR4) is a transmembrane protein and the receptor for lipopolysaccharides (LPS). Downregulated expression of TLR4 can reduce acute liver injure [9]. The expression of PCNA, IL-2, and TLR4 play a role in liver regeneration and immune response in ALF.

Jiedu Huayu (JDHY) granules are a traditional Chinese medicine prescription that has been used for over two decades to treat ALF. In our previous clinical studies, we showed that JDHY had hepatic-protective effects in hepatitis B-related acute on chronic liver failure patients and improved liver function and reduced complications [10]. Moreover, animal experiments demonstrated that JDHY decreased the expression of caspase-3 mRNA in an ALF rat model, suggesting JDHY may be an effective therapeutic for treating hepatocyte-related apoptosis [11]. The effects of JDHY on ALF are still unknown. In this study, we determined the effects of JDHY granules on ALF and investigated the role of JDHY on the expression of PCNA, IL-2, and TLR4.

## 2. Methods

### 2.1. Drug Preparation

**Figure 1:**
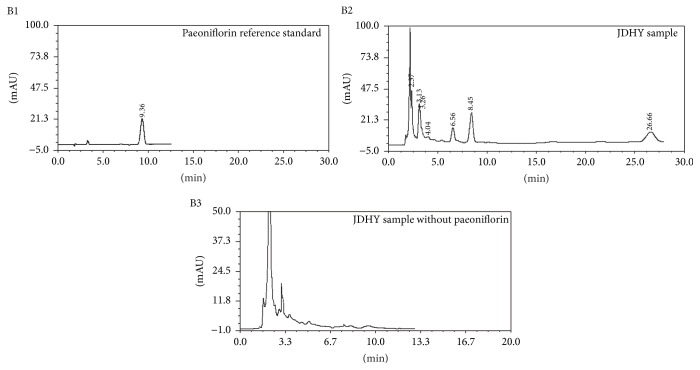
Paeoniflorin was used as a quality control and analyzed by HPLC (B1–B3).

Traditional Chinese herb granules manufactured by* Jiangyin Tian Jiang* Pharmaceutical Co. Ltd. (Jiangsu, China) were purchased from the First Affiliated Hospital of Guangxi University of Traditional Chinese Medicine. The components included in the the JDHY granules are listed in Table 1. For quality control purposes [12], paeoniflorin, a representative component of herbal medicines, was determined by high performance liquid chromatography (HPLC). The results are shown in Figure 1. Prior to usage, 4 g of dry herbal mixture was resuspended in 1 ml of distilled water.

### 2.2. Animal Studies

Animal experiments were approved by the animal care and use committee of Guangxi Traditional Chinese Medicine University. Male Wistar rats, weighing 200 ± 20 g, were purchased from the animal experimental center of Guangxi Medical University (Nanning, China) and were from a pathogen-free environment. All rats were fed with a specific pathogen-free (SPF) diet. D-Galactosamine (D-GalN) and lipopolysaccharide (LPS) were purchased from Tianjin Chemical Co. Ltd. (Sigma, MO, USA) and were used to induce liver injury [13].

Rats (*n* = 55) were randomized into four groups: (1) blank control group (control, *n* = 10) given distilled water via gavage, (2) model group (model, *n* = 15) injected intraperitoneally with D-GalN and LPS, (3) JDHY granules-low dose group (low dose, *n* = 15) injected intraperitoneally with D-GalN and LPS and given JDHY 4.4 g/kg/d via gavage, and (4) JDHY granules-high dose group (high dose, *n* = 15) injected intraperitoneally with D-GalN and LPS and given JDHY 8.8 g/kg/d via gavage. JDHY granules were given via gavage for 3 days before D-GalN and LPS were administered in the low dose and high dose groups. The injection doses of D-GalN and LPS were 900 mg/kg and 10 *μ*g/kg, respectively [14].

Twelve hours after D-GalN and LPS administration, 3 ml of blood was collected from live rats (control group, *n* = 10; model group, *n* = 9; low dose group, *n* = 11; high dose group, *n* = 12) for detection of aminotransferase (ALT), aspartate aminotransferase (AST), total bilirubin (TBIL), and prothrombin time (PT). After blood collection, rats were sacrificed and the left lobe of the liver was collected for further studies, such as proliferating cellular nuclear antigen (PCNA) immunohistochemical assays, quantitative reverse transcription polymerase chain reaction (qRT-PCR), and Western Blot.

### 2.3. Serum ALT, AST, TBIL, and PT Detection

For the preparation of serum, whole blood was collected and centrifuged at 2000 rpm for 15 min at 4°C. The supernatant, which designated serum, was transferred to a clean tube. Serum was used to measure levels of ALT, AST, TBIL, and prothrombin time (PT), which are frequently used to reflect liver function. Measurements were performed by an automatic biochemical analyzer in the Department of Laboratory Medicine, The First Affiliated Hospital of Guangxi University of Traditional Chinese Medicine (Nanning, China).

### 2.4. Survival Studies

In an additional study, 115 rats were divided into (1) control group, *n* = 10; (2) model group, *n* = 35; (3) low dose group, *n* = 35; and (4) high dose group, *n* = 35. Treatments were as mentioned before. Survival was determined at 48 hours after administration of LPS and D-GalN.

### 2.5. Immunohistochemical Analysis of PCNA Expression

Liver tissues were fixed for 24 h in 10% formalin, embedded in paraffin, and cut into 4 *μ*m sections. Each section was incubated overnight at 4°C with an anti-PCNA antibody (1 : 500 dilution, Santa Cruz Biotechnology, USA) followed by incubation with a horseradish peroxidase- (HRP-) conjugated goat anti-mouse antibody (Dako, Denmark). A diaminobenzidine (DAB) kit (Dako, Denmark) was used for immunohistochemical visualization. Sections were visualized at 200x magnification, and PCNA staining intensity was analyzed in six random fields per section using Image-pro Plus 6.0 software. Each sample was analyzed in triplicate.

### 2.6. IL-2 and TLR4 mRNA Expression

TRIzol reagent (Invitrogen, China) was used to isolate total RNA from liver samples derived from rats that underwent the different treatments. The SuperScriptIII Reverse Transcriptase (Invitrogen, China) was used to synthesize cDNA, which was then amplified using a SYBR Green qPCR Mix (Invitrogen, China) and specific primers using a PTC-100 qPCR System (MJ Research, USA). Primer sequences are listed in Table 2. The housekeeping gene actin was used as an internal control. Target gene levels are presented as a ratio of levels in treated tissues to levels detected in control tissues according to the 2^−ΔΔCt^ method [15].

### 2.7. Western Blot Analysis of IL-2 and TLR4

Liver tissue was homogenized in ice-cold loading buffer (pH 6.8). Total protein was extracted using RIPA Lysis Buffer (Beyotime Biotechnology, China) and analyzed using a BCA protein analysis kit. Proteins (40 *μ*g) were separated using a 13% SDS-PAGE gel and transferred onto a nitrocellulose membrane for Western blot analysis. Membranes were blocked in 5% bovine serum albumin and probed with antibodies against IL-2 and TLR4 (1 : 500 dilution, Santa Cruz Biotechnology, USA). After washing with PBST, membranes were incubated with HRP-conjugated goat anti-mouse IgG (1 : 5000 dilution, KPL, USA). After washing with PBST, membranes enhanced chemiluminescent substrate was applied before membranes were being exposed to film. Images were taken and quantified using Image-pro Plus 6.0 software.

### 2.8. Statistical Methods

Data were subjected to statistical analysis using the statistical package for social sciences (SPSS), version 18.00. A one-way ANOVA or Student's *t*-test was used to determine statistical significance. Survival was assessed using Kaplan-Meier survival analysis and analyzed using the Log-rank test. A probability of *P* < 0.05 was considered statistically significant. All data are presented as mean ± SD.

## 3. Results

### 3.1. Serum ALT, AST, TBIL, and PT Levels

**Figure 2:**
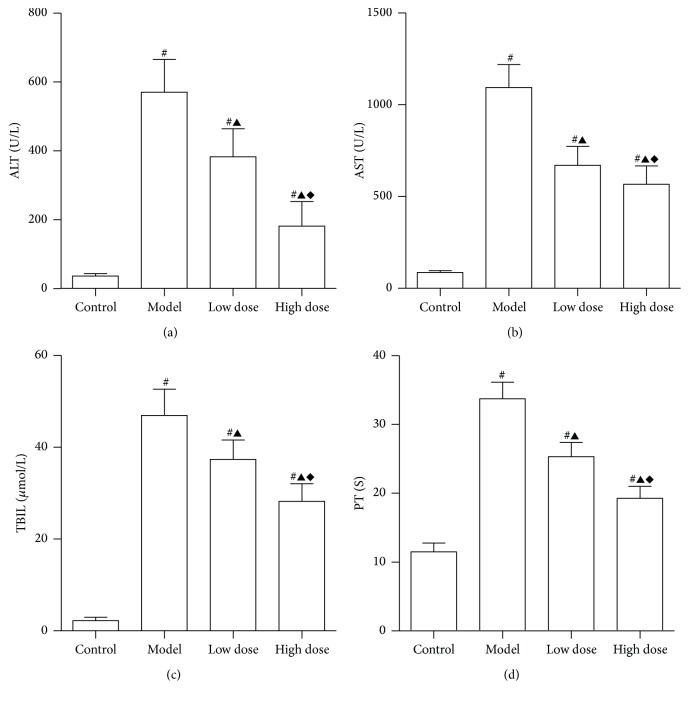
Serum levels of ALT, AST, TBIL, and PT after treatment. (a) ALT; (b) AST; (c) TBIL; (d) PT. Control: negative control, rats were given with distilled water via gavage; model: rats injected with LPS + D-GalN; low dose: rats injected with LPS + D-GalN and treated with a low dose of JDHY; high dose: rats injected with LPS + D-GalN and treated with a high dose of JDHY. For control, model, low dose, and high dose groups, the ALT levels were 37.45 ± 7.01 U/L, 571.47 ± 95.18 U/L, 383.46 ± 82.41 U/L, and 182.64 ± 71.34 U/L, respectively. AST levels were 88.93 ± 10.23 U/L, 1097.43 ± 124.66 U/L, 673.16 ± 102.13, and 569.32 ± 100.57, respectively. TBIL levels were 2.36 ± 0.78 *μ*mol/L, 47.11 ± 5.74 *μ*mol/L, 37.55 ± 4.23 *μ*mol/L, and 28.36 ± 3.85 *μ*mol/L, respectively. PT was 11.53 ± 1.24 s, 33.78 ± 2.41 s, 25.36 ± 2.04 s, and 19.32 ± 1.74 s, respectively. For (a), (b), (c) and (d), ^#^*P* < 0.05 compared to the control group, ^▲^*P* < 0.05 compared to the model group, and ^*◆*^*P* < 0.05 compared to the low dose group.

Serum levels of ALT, AST, TBIL, and PT are shown in Figure 2. Our data shows that, compared to the control group, LPS + D-GalN injection significantly increases levels of ALT, AST, and TBIL. In addition, PT time was extended when compared to the control group (*P* < 0.05). Following treatment with low and high doses of JDHY, liver function was improved as indicated by reduced levels of ALT, AST, TBIL, and PT time when compared to the model group (*P* < 0.05). Moreover, high doses of JDHY showed better treatment effects as compared to the low dose group (*P* < 0.05). Together, these parameters are indicative of severe liver damage.

### 3.2. Survival Analysis

**Figure 3:**
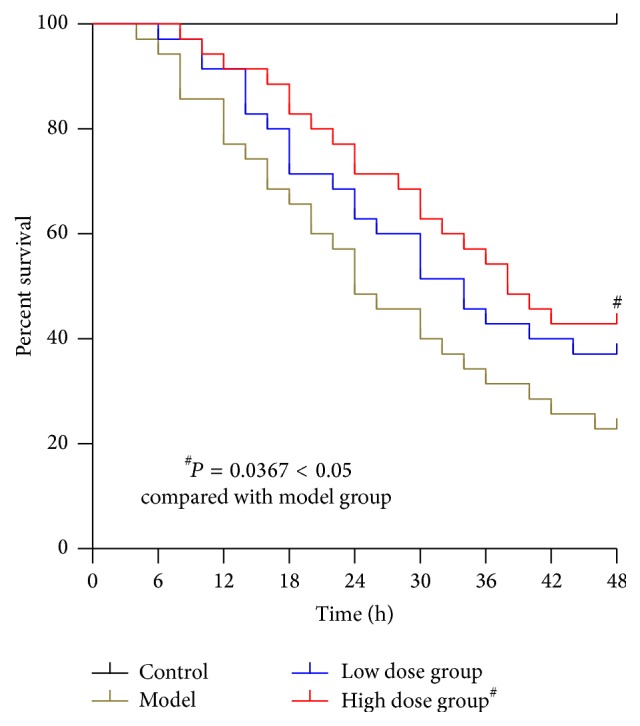
Survival studies. Control: negative control given distilled water via gavage; model: rats injected with LPS + D-GalN; low dose: rats injected with LPS + D-GalN and treated with a low dose of JDHY; high dose: rats injected with LPS + D-GalN and treated with a high dose of JDHY. ^#^*P* < 0.05 compared to the model group.

In this study, none of the rats in the control group died.  Figure 3 shows that, in rats in which ALF was induced by administration of LPS and D-GalN, high doses of JDHY significantly improved survival when compared to the model group (*P* < 0.05).

### 3.3. PCNA Immunohistochemistry

**Figure 4:**
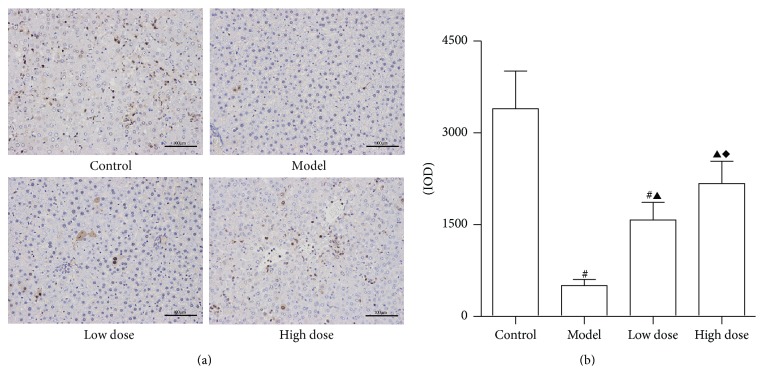
PCNA immunohistochemistry. (a) PCNA immunohistochemistry; (b) PCNA positive rates per group. Control: negative control given distilled water via gavage; model: rats injected with LPS + D-GalN; low dose: rats injected with LPS + D-GalN and treated with a low dose of JDHY; high dose: rats injected with LPS + D-GalN and treated with a high dose of JDHY. The PCNA positive (light density of positive cumulative, IOD) rates were 3394.51 ± 614.63, 502.81 ± 102.67, 1576.84 ± 288.27, and 2172.43 ± 363.60 in control, model, low dose, and high dose groups, respectively. ^#^*P* < 0.05 compared to the control group, ^▲^*P* < 0.05 compared to the model group, and ^*◆*^*P* < 0.05 compared to the low dose group.

The light density of PCNA positive cumulative of rats in the control group was significantly increased when compared to other groups (Figure 4), indicating that the regeneration rate in normal liver cells is higher than that of other groups. The PCNA positive rates were significantly decreased in the model group when compared to the control group (*P* < 0.05). Our data shows that high doses of JDHY can improve PCNA positive rates when compared to the model group and low dose group (*P* < 0.05). Together, these results suggest that JDHY treatment promotes hepatocyte regeneration in ALF in rats.

### 3.4. mRNA and Protein Expression of IL-2

**Figure 5:**
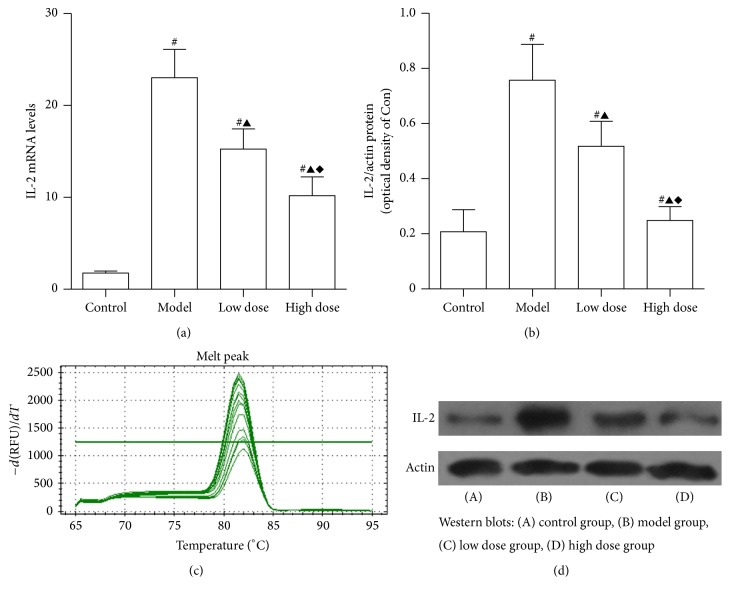
mRNA and protein expression levels of IL-2. (a) IL-2 mRNA expression, (b) IL-2 protein expression, (c) amplification curves, and (d) Western blot analysis. Control: negative control given distilled water via gavage; model: rats injected with LPS + D-GalN; low dose: rats injected with LPS + D-GalN and treated with a low dose of JDHY; high dose: rats injected with LPS + D-GalN and treated with a high dose of JDHY. For control, model, low dose, and high dose groups, the IL-2 mRNA expression levels were 1.83 ± 0.24, 23.12 ± 3.07, 15.33 ± 2.21, and 10.25 ± 2.06 and the protein expression levels were 0.21 ± 0.08, 0.76 ± 0.13, 0.52 ± 0.09, and 0.25 ± 0.05, respectively. ^#^*P* < 0.05 compared to control group, ^▲^*P* < 0.05 compared to model group, and ^*◆*^*P* < 0.05 compared to the low dose group.

IL-2 is one of the immune regulators that is involved in acute liver injury. As shown in Figure 5(a), a substantial increase in mRNA and protein levels of IL-2 expression were observed in the model group as compared to the control group (*P* < 0.05). Following treatment with JDHY and high doses of JDHY, IL-2 mRNA and protein levels dramatically decreased in the high dose group compared to the model and low dose group (*P* < 0.05).

### 3.5. mRNA and Protein Expression of TLR4

**Figure 6:**
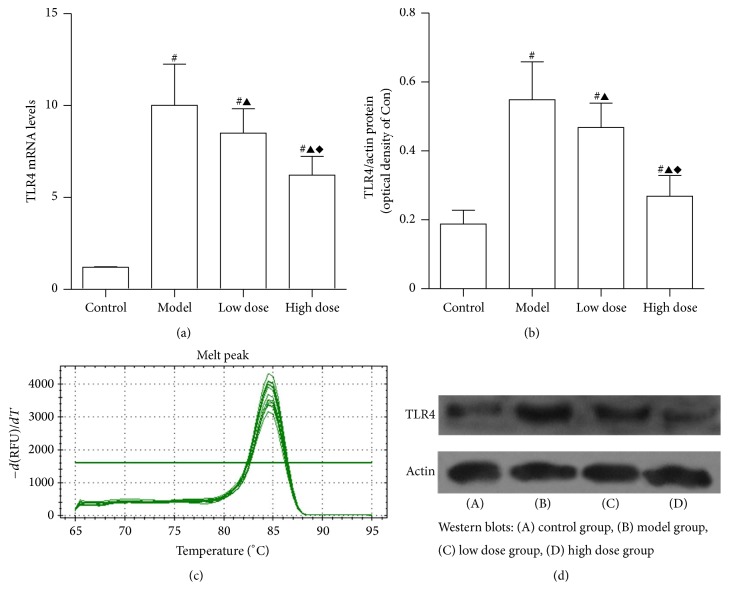
mRNA and protein expression levels of TLR4. (a) TLR4 mRNA expression levels, (b) TLR4 protein expression levels, (c) amplification curves, and (d) Western blot analysis. Control: negative control given distilled water via gavage; model: rats injected with LPS + D-GalN; low dose: rats injected with LPS + D-GalN and treated with a low dose of JDHY; high dose: rats injected with LPS + D-GalN and treated with a high dose of JDHY. For control, model, low dose, and high dose groups, the TLR4 mRNA expression levels were 1.21 ± 0.02, 10.02 ± 2.23, 8.51 ± 1.32, and 6.22 ± 1.02. Protein expression levels were 0.19 ± 0.04, 0.55 ± 0.11, 0.47 ± 0.07, and 0.27 ± 0.06, respectively. ^#^*P* < 0.05 compared to control group, ^▲^*P* < 0.05 compared to model group, and ^*◆*^*P* < 0.05 compared to the low dose group.

TLR4 is a primary receptor that mediates cytokine release and tissue damage in various conditions.  Figure 6 shows that, after administration of LPS and D-GalN, TLR4 mRNA and protein levels were increased when compared to the control group (*P* < 0.05). Moreover, JDHY treatment significantly reduced the expression of TLR4 in a dose-responsive manner (*P* < 0.05).

## 4. Discussion

Combined administration of LPS and D-GalN is a commonly used approach to construct a rat model of ALF [16]. In our study, an increase in serum levels of ALT, AST, and TBIL increased and an elongated PT was observed. In addition, rats died rapidly following LPS and D-GalN injection, which indicated that an ALF model was successfully established. After treatment with JDHY granules, liver function improved and survival times extended. High dose JDHY granules showed the strongest treatment effect.

The JDHY granules used in this study have been used as a treatment method in the First Affiliated Hospital of GuangXi University of Traditional Chinese Medicine for many years. Clinical trials have also shown the protective effects of JDHY granules on ALF [10]. The active ingredient of this Chinese medicine is* Artemisia capillaris*, which has been well recognized as a herb with therapeutic efficacy in liver diseases. Therefore, JDHY is a widely used therapeutic in, for example, antihepatic fibrosis where it functions by increasing antioxidant activities and reducing extracellular matrix protein production [17, 18].* Radix paeoniae rubrathe* was found to relax vascular smooth muscles by regulating Akt and SOCE-eNOS-cGMP-mediated pathways [19]. It also protects against liver damage by inducing CCl_4_ that correlates with antioxidant and free radical scavenger effects [20].* Rheum officinale* was found to reduce inflammation in the serum of ALF mice by regulating NF-*κ*B signaling pathways and the expression of proteins against apoptosis [21].* Oldenlandia diffusa* inhibits inflammation via suppression of caspase-1 activation in mouse peritoneal macrophages [22].* Radix curcumae* was found to reduce proinflammatory cytokines by blocking NF-*κ*B signal pathways [23]; however, it was also found to regulate cell proliferation and apoptosis [24].* Acorus gramineus* was found to have a therapeutical role in disorders of the central nervous system such as convulsions and epilepsy [25]. JDHY granules are a combination of these Chinese herb medicines and are suggested to clear heat, disintoxication, and resolving stasis. In a previous clinical trial, we demonstrated that JDHY granules can improve hepatic function in ALF [10]; however, the potential mechanisms involved are still unclear.

TLR4, a transmembrane protein, is composed of 22 leucine-rich repeats in the extracellular domain conferring a horseshoe-like shape on the protein [26]. In 1998, TLR4 was identified as a LPS receptor [27]. TLR4 can activate downstream pathways of MyD88/IRAK and TRIF/IKK*ε*/TBK1 that are involved in the innate immune response [28]. TLR4 is primarily present in Kupffer cells in the liver and plays an important role in activation of the inflammatory cytokine cascade. In addition, TLR4 has been found to have a pivotal role in the progression of acute liver injury [29]. It has previously been demonstrated that downregulation of TLR4-related pathways has a therapeutic effect in ALF models [30, 31]. In our study, we found that both mRNA and protein expression levels of TLR4 were remarkably increased in our model group compared to control group. In addition, treatment with JDHY granules significantly decreased TLR4 expression especially in the high dose JDHY granules treatment group. These results indicate that JDHY granules can improve liver function in ALF rats by inhibiting TLR4-related pathways.

IL-2 is a glycoprotein with a molecular weight of ~15.5 Kd. The mature IL-2 molecule is composed of 133 amino acid residues [32]. IL-2 is mainly secreted by activated T lymphocyte and is hardly expressed when cells are in rest. T cell receptor (TCR) is an important marker on T lymphocytes, which, combined with CD3, forms a TCR-CD3 complex on the cell surface [33]. In ALF, the TCR immune signaling cascade is activated through CD3 transmission after recognition of the antigen, which causes IL-2 to be largely expressed by CD_4_^+^ and CD_8_^+^ T lymphocytes [34]. In acute liver injury, the expression of IL-2 is significantly increased [35]. In this study, the expression of IL-2 was extremely high in our model group when compared to the control group. After treatment with JDHY granules, mRNA and protein expression levels of IL-2 were downregulated. This inhibitory effect was further increased when a high dose of JDHY granules was used. Therefore, treatment with JDHY granules improves liver function in an acute model of rat liver failure by downregulating TLR4 and IL-2 pathways.

It is well accepted that the liver has a strong regenerative capability [36]. PCNA can directly participate in DNA synthesis. Therefore, PCNA may be an indicator for evaluating liver regeneration in ALF [4]. In this study, the PCNA positive rates were significantly higher in the high dose JDHY granules group when compared to all other groups, which indicates the high regeneration rates that are caused by JDHY granules. However, whether the promotion of regeneration was directly induced by JDHY granules or by suppression of subsequent inflammatory effects remains to be investigated. Further studies will be needed to investigate the relation between inflammation and regeneration during the treatment of ALF.

In this study, behavioral changes such as changes in appetite and response to stimulation were also investigated. We found that, in an acute rat liver failure model, appetite and behavior were significantly decreased and were accompanied by the development of a series of symptoms including yellow urine, lethargy, listlessness, and convulsion. However, treatment with JDHY granules decreased these symptoms remarkably and more importantly no treatment-related adverse events were observed.

## 5. Conclusion

High doses of JDHY granules improve liver function and survival in rats with ALF. These therapeutic effects are associated with a reduction in inflammatory events and promotion of hepatocyte regeneration.

## Figures and Tables

**Table 1:** Composition of JDHY granules.

Components	Amount used (g)	Lot number
*Artemisia capillaries*	4	1508091
*Radix paeoniae rubrathe*	7.5	1507064
*Rheum officinale*	5	1506076
*Oldenlandia diffusa*	2	1509108
*Radix curcumae*	1.5	1507074
*Acorus gramineus*	3	1507038
Total	23	

**Table 2:** Primer sequences used in quantitative RT-PCR.

Parameters	5′ primer sequence	3′ primer sequence
IL-2	CCAAGCAGGCCACAGAATTG	TCCAGCGTCTTCCAAGTGAA
TLR4	CCAGGAAGGCTTCCACAAGA	AATTCGACCTGCTGCCTCAG
Actin	GCTCAGGAGGAGCAATGATCTTG	GTACGCCAACACAGTGCTGTC
